# Hypergastrinemia is associated with an increased risk of gastric adenocarcinoma with proximal location: A prospective population‐based nested case‐control study

**DOI:** 10.1002/ijc.33354

**Published:** 2020-11-02

**Authors:** Eivind Ness‐Jensen, Erling Audun Bringeland, Fredrik Mattsson, Patricia Mjønes, Jesper Lagergren, Jon Erik Grønbech, Helge Lyder Waldum, Reidar Fossmark

**Affiliations:** ^1^ HUNT Research Centre, Department of Public Health and Nursing NTNU, Norwegian University of Science and Technology Levanger Norway; ^2^ Department of Medicine Levanger Hospital, Nord‐Trøndelag Hospital Trust Levanger Norway; ^3^ Upper Gastrointestinal Surgery, Department of Molecular Medicine and Surgery Karolinska Institutet, Karolinska University Hospital Stockholm Sweden; ^4^ Department of Gastrointestinal Surgery St. Olav's Hospital, Trondheim University Hospital Trondheim Norway; ^5^ Department of Clinical and Molecular Medicine NTNU, Norwegian University of Science and Technology Trondheim Norway; ^6^ Department of Pathology, St. Olav's Hospital Trondheim University Hospital Trondheim Norway; ^7^ School of Cancer and Pharmaceutical Sciences King's College London London UK; ^8^ Department of Gastroenterology and Hepatology St. Olav's Hospital, Trondheim University Hospital Trondheim Norway

**Keywords:** cancer, epidemiology, gastrin, Lauren, stomach

## Abstract

The incidence of proximal gastric adenocarcinoma is increasing among younger adults. Rodent models have shown that hypergastrinemia causes carcinogenesis in the proximal stomach. The aim of our study was therefore to assess if hypergastrinemia was associated with an increased risk of developing gastric adenocarcinoma also in humans. A prospective population‐based nested case‐control study within the Nord‐Trøndelag Health Study (HUNT) cohort, Norway, was used to assess this association. Serum was collected from 78 962 participants in 1995 to 1997 and 2006 to 2008. In the cohort, 181 incident gastric adenocarcinoma cases were identified from the Norwegian Cancer and Patient Registries through 2015 and matched with 359 controls. The risk of gastric adenocarcinoma was compared between participants with prediagnostic hypergastrinemia (>60 pmol/L) and normal serum gastrin (≤60 pmol/L). Logistic regression provided odds ratios (ORs) with 95% confidence intervals (CIs), adjusted for body mass index, tobacco smoking and comorbidity. Hypergastrinemia was associated with increased risk of gastric adenocarcinoma overall (OR 2.2, 95% CI 1.4‐3.4) and in particular for gastric adenocarcinoma with proximal location (OR 6.1, 95% CI 2.7‐13.8), but not with gastric adenocarcinoma with distal location (OR 1.7, 95% CI 0.9‐3.4). Moreover, hypergastrinemia was associated with an increased risk of gastric adenocarcinoma of intestinal histological type (OR 3.8, 95% CI 1.8‐7.9), but not for diffuse histological type (OR 1.6, 95% CI 0.7‐3.7). In conclusion, hypergastrinemia was associated with an increased risk of proximal and intestinal type gastric adenocarcinoma.

## Abstract 2

**What's new?**

The incidence of proximal gastric adenocarcinoma has been reported to increase among younger adults in Western countries. Rodent models have shown that serum gastrin levels above the normal range cause carcinogenesis in the proximal stomach. In this first prospective population‐based study on the association between hypergastrinemia and gastric adenocarcinoma, the risk of gastric adenocarcinoma in the proximal stomach, but not in the distal stomach, was markedly increased in hypergastrinemic individuals. The finding supports the hypothesis that hypergastrinemia mediates the development of gastric adenocarcinoma in the proximal stomach, where mucosal proliferation is stimulated by gastrin.

**Abbreviations**
CI: confidence interval
ECL: enterochromaffin‐like
HUNT: Nord‐Trøndelag Health Study
H pylori: *Helicobacter pylori*
ICD‐O: International Classification of Diseases for Oncology
IQR: interquartile range
OR: odds ratio
PPI: proton pump inhibitor

## 1 INTRODUCTION

Gastric adenocarcinoma is the fifth most common cancer worldwide.^1^ The main risk factor for non‐cardia gastric adenocarcinoma is atrophic gastritis due to infection with *Helicobacter pylori*.^2^, ^3^, ^4^ In Western populations, the incidence of gastric adenocarcinoma has declined during the last decades, which is probably mainly due to the decreasing prevalence of *H pylori* infections and subsequently declined incidence of gastric adenocarcinoma of the intestinal histological type (Laurén's classification).^5^ However, recent studies have shown increasing incidence of gastric adenocarcinoma in the proximal stomach in younger cohorts (<50 years of age) in Western countries, such as the United States and the United Kingdom.^6^, ^7^, ^8^ Suggested reasons for this increase include increasing prevalence of autoimmune gastritis and long‐term use of proton pump inhibitors (PPIs), both causing hypergastrinemia.^9^, ^10^, ^11^, ^12^

The hormone gastrin is the main stimulator of gastric acid secretion and oxyntic mucosal growth.^13^ Gastrin is released by the antral G‐cells in the distal stomach in response to elevation of intragastric pH and protein‐containing meals.^14^, ^15^ Subsequently, gastrin stimulates enterochromaffin‐like (ECL) cells of the oxyntic mucosa in the proximal stomach to secrete histamine, which in turn stimulates the parietal cells to secrete acid.^16^ Gastritis causing atrophy of the oxyntic mucosa leads to reduction in acid secretion, and the resulting loss of negative feedback on gastrin release leads to hypergastrinemia.

Hypergastrinemia increases the risk of gastric neoplasia in numerous animal models, suggesting that the carcinogenic effects of *H pylori*, autoimmune gastritis and PPIs may be mediated by hypergastrinemia.^17^, ^18^, ^19^, ^20^, ^21^, ^22^, ^23^, ^24^ In patients with hypergastrinemia, gastric adenocarcinomas are overrepresented in the corpus,^25^ and the risk of gastric adenocarcinoma might progressively increase with the degree of oxyntic mucosal gastritis.^26^

The aim of our study was to test the hypothesis that hypergastrinemia is associated with development of gastric adenocarcinoma and particularly so in the proximal stomach.

## 2 METHODS

### 2.1 Design

This was a population‐based nested case‐control study within the Nord‐Trøndelag Health Study (HUNT). In HUNT, all residents in Nord‐Trøndelag County, Norway, from 20 years of age were invited to participate in repeated health surveys. The second and third surveys were performed 15 August 1995 to 18 June 1997 (HUNT2) and 3 October 2006 to 25 June 2008 (HUNT3). The surveys collected data from questionnaires regarding health‐related factors and standardized physical examinations. Blood samples were also collected and stored at −80°C from 65 237 (response rate 69.5%) and 50 807 (54.1%) individuals, respectively.^27^

The Cancer Registry of Norway has since 1951 and the Norwegian Patient Registry has since 2008 collected data on all cancers in Norwegian residents. Using the unique 11‐digit person identification number assigned to every Norwegian resident, HUNT data were linked to the Cancer Registry, to the Patient Registry and to medical records at the treating hospitals (Nord‐Trøndelag Hospital Trust and St. Olav's Hospital) until 31 December 2015.

### 2.2 Gastric adenocarcinoma cases

HUNT2 and HUNT3 participants were followed up from their participation date in the respective HUNT study until 31 December 2015. Newly diagnosed gastric cancer during follow‐up was identified based on the third version of the International Classification of Diseases for Oncology (ICD‐O‐3: C16). Participants with any previous or prevalent gastric cancer were excluded. The location of the cancers was defined through review of the medical records at the treating hospitals. This was performed by two senior upper gastrointestinal surgeons (EAB and JEG) and one senior gastroenterologist (ENJ). Location was categorized as proximal (corpus, fundus or Siewert's cardia type III^28^), distal (antrum), indefinite or unknown. Siewert's cardia type I and II and gastric stump cancers were excluded. The histological type (adenocarcinoma) and a uniform histological classification according to Laurén (diffuse, intestinal, mixed and indeterminate)^29^ were secured by a pathologist (PM). All other histological types than adenocarcinomas were excluded. The reviewers were blinded to serum gastrin values.

### 2.3 Controls

The controls were randomly selected from the remaining HUNT2 and HUNT3 participants. For each case, two age‐ (within 1 year), sex‐ and sample date‐ (within 6 months) matched controls without any cancer were selected.

### 2.4 Serum gastrin

Serum gastrin was analyzed on the stored prediagnostic serum samples collected from the cases and controls as they participated in HUNT. If a case participated in both HUNT2 and HUNT3 before the gastric adenocarcinoma developed, the gastrin level was determined by the most recent serum sample.

The gastrin analyses were performed using a radioimmunoassay kit (Euria Gastrin MD302, Euro Diagnostica, Malmö, Sweden), which is reported to have superior diagnostic accuracy among the available kits.^30^ With this kit, hypergastrinemia was defined as values >60 pmol/L. The gastrin analyses were run as two parallel analyses from each sample, and the mean value from these two was used in the statistical analyses.

### 2.5 Confounders

The possible confounding factors assessed were age, sex, body mass index, tobacco smoking and comorbidity. The confounders were assessed as part of the HUNT surveys at the time of blood sampling. Body mass index was assessed based on objective measures of weight and height performed by trained personnel. Tobacco smoking status was assessed based on self‐reported questionnaires of smoking habits. Comorbidity was assessed based on self‐reported presence of chronic obstructive pulmonary disease, cardiovascular disease (including myocardial infarction, angina pectoris and stroke), diabetes mellitus and hypertension.

### 2.6 Statistical analyses

Descriptive data are presented as absolute numbers, percentages, mean (standard deviation [SD]) and median (interquartile range [IQR]). As this was a matched design, the association between hypergastrinemia and gastric adenocarcinoma was assessed by conditional multivariable logistic regression, providing odds ratios (ORs) and 95% confidence intervals (CIs), adjusted for age (continuous), sex, body mass index (<25, 25‐30 or ≥30 kg/m^2^), tobacco smoking (never, previous or current) and comorbidity (absent or present). Analyses were also performed by tumor location (proximal, distal or indefinite/unknown) and histological classification according to Laurén (diffuse or intestinal). To evaluate whether the association between hypergastrinemia and gastric adenocarcinoma was modified by age (< or ≥median age), sex or BMI (<30 or ≥30 kg/m^2^), an interaction term with the main exposure was included one by one in the fully adjusted model and ORs were derived within each stratum for age, sex and BMI. Due to low partial missing for each variable in the model, only complete case analyses were performed. An experienced biostatistician (FM) conducted all data management and statistical analyses according to a predefined study protocol.

## 3 RESULTS

### 3.1 Characteristics of gastric adenocarcinoma cases

Among the 78 962 unique participants in the cohort, 181 (0.2%) individuals developed gastric adenocarcinoma during follow‐up and 359 participants were selected as controls. Median time (IQR) from baseline to diagnosis was 5.7 (3.0‐9.1) years for all cases. The characteristics of the cases and the controls are shown in Table 1. The tumor location was proximal in 81 (45%), distal in 70 (39%), indefinite in 27 (15%) and unknown in 3 (2%). The tumor histology was diffuse in 63 (35%), intestinal in 74 (41%), mixed in 15 (8%), indeterminate in 22 (12%) and unknown (no biopsy) in 7 (4%). The median serum gastrin concentration was higher in cases than controls (58 pmol/L vs 48 pmol/L) and hypergastrinemia was more prevalent among cases than controls (47% vs 33%). The median age was 72 years (range 45‐91 years) and 85 (47%) were women. More cases than controls were ever smokers, but there were only minor differences between the cases and controls in the other possible confounding factors.

**TABLE 1:** Characteristics of cases with gastric adenocarcinoma and matched controls (1:2)

		Cases (No. 181)	Controls (No. 359)^a^
Tumor location	No. (%)	181 (100.0)	
Proximal^b^	No. (%)	81 (44.8)	
Distal^c^	No. (%)	70 (38.7)	
Indefinite	No. (%)	27 (14.9)	
Unknown	No. (%)	3 (1.7)	
Laurén's histological type			
Diffuse	No. (%)	63 (34.8)	
Intestinal	No. (%)	74 (40.9)	
Mixed	No. (%)	15 (8.3)	
Indeterminate	No. (%)	22 (12.2)	
Unknown	No. (%)	7 (3.9)	
Time of diagnosis, y			
1995 to 2000	No. (%)	45 (24.9)	
2001 to 2005	No. (%)	47 (26.0)	
2006 to 2010	No. (%)	44 (24.3)	
2011 to 2015	No. (%)	45 (24.9)	
Follow‐up time^d^, y	Mean (SD)	6.7 (4.5)	
	Median (IQR)	5.7 (3.0–9.1)	
Serum gastrin^e^, pmol/L	Mean (SD)	109.3 (143.7)	74.5 (109.0)
	Median (IQR)	58.2 (41.3‐97.2)	48.0 (35.0‐68.0)
	≤60, No. (%)	95 (52.5)	241 (67.1)
	>60, No. (%)	85 (47.0)	117 (32.6)
	Missing, No. (%)	1 (0.6)	1 (0.3)
Age^e^, years	Mean (SD)	70.6 (10.4)	70.5 (10.5)
	Median (IQR)	72.2 (64.6‐78.2)	72.3 (64.6‐78.0)
Sex^e^	Women, No. (%)	85 (47.0)	170 (47.4)
	Men, No. (%)	96 (53.0)	189 (52.6)
Body mass index^e^	Mean (SD)	27.4 (4.0)	27.1 (4.2)
	Median (IQR)	26.9 (24.7‐29.9)	26.5 (24.3‐29.0)
	<25.0, No. (%)	51 (28.2)	112 (31.2)
	25.0‐29.9, No. (%)	87 (48.1)	171 (47.6)
	≥30.0, No. (%)	41 (22.7)	72 (20.1)
	Missing, No. (%)	2 (1.1)	4 (1.1)
Tobacco smoking status^e^	Never, No. (%)	55 (30.4)	164 (45.7)
	Previous, No. (%)	81 (44.8)	114 (31.8)
	Current, No. (%)	39 (21.5)	69 (19.2)
	Missing, No. (%)	6 (3.3)	12 (3.3)
Chronic obstructive pulmonary disease^e^	Absent, No. (%)	162 (89.5)	318 (88.6)
	Present, No. (%)	19 (10.5)	41 (11.4)
Cardiovascular disease^e^	Absent, No. (%)	143 (79.0)	280 (78.0)
	Present, No. (%)	37 (20.4)	78 (21.7)
	Missing, No. (%)	1 (0.6)	1 (0.3)
Diabetes mellitus^e^	Absent, No. (%)	165 (91.2)	336 (93.6)
	Present, No. (%)	15 (8.3)	21 (5.8)
	Missing, No. (%)	1 (0.6)	2 (0.6)
Hypertension^e^	Absent, No. (%)	123 (68.0)	246(68.5)
	Present, No. (%)	57 (31.5)	110 (30.6)
	Missing, No. (%)	1 (0.6)	3 (0.8)

Abbreviations: IQR, interquartile range.

^a^ Only one control available for three distal cases, two diffuse types and one indeterminate type.

^b^ Corpus, fundus, Siewert cardia type III.

^c^ Antrum.

^d^ From date of participation in the Nord‐Trøndelag Health Study to the date of cancer diagnosis.

^e^ Assessed at baseline at participation in the Nord‐Trøndelag Health Study.

### 3.2 The risk of gastric adenocarcinoma by location

Hypergastrinemia was associated with an increased risk of gastric adenocarcinoma overall (OR 2.2, 95% CI 1.4‐3.4) (Table 2). The association was stronger in patients with proximal tumor location (OR 6.1, 95% CI 2.7‐13.8), but weaker and statistically nonsignificant in patients with distal tumor location (OR 1.7, 95% CI 0.9‐3.4). The risk of proximal tumor location associated with hypergastrinemia was seemingly higher among women (OR 14.3, 95% CI 3.6‐57.5) than men (OR 3.6, 95% CI 1.4‐9.6). There was no effect modification by age or body mass index (results not shown).

**TABLE 2:** The odds ratio (OR) with 95% confidence interval (CI) of gastric adenocarcinoma with hypergastrinemia (serum gastrin >60 pmol/L) by tumor location and sex

	Both sexes								Women				Men			
	Model 1^a^				Model 2^b^				Model 2^b^				Model 2^b^			
	Cases (No.)^c^	Ctrls (No.)^d^	OR	95% CI	Cases (No.)^c^	Ctrls (No.)^d^	OR	95% CI	Cases (No.)^c^	Ctrls (No.)^d^	OR	95% CI	Cases (No.)^c^	Ctrls (No.)^d^	OR	95% CI
All locations	174	328	2.0	1.4‐3.1	174	328	2.2	1.4‐3.4	82	151	2.4	1.3‐4.5	92	177	2.0	1.1‐3.7
Proximal^e^	74	138	3.5	1.8‐6.8	74	138	6.1	2.7‐13.8	33	59	14.3	3.6‐57.5	41	79	3.6	1.4‐9.6
Distal^f^	70	136	1.6	0.9‐3.1	70	136	1.7	0.9‐3.4	35	68	1.8	0.7‐4.2	35	68	1.7	0.6‐4.6
Indefinite/unknown	30	54	0.7	0.3‐2.2	30	54	0.7	0.2‐2.2	14	24	0.6	0.1‐3.0	16	30	0.8	0.1‐4.3

^a^ Matched for sex, age and sample time.

^b^ As model 1 and adjusted for body mass index, tobacco smoking status and comorbidity.

^c^ Number of cases included in the analysis (with complete data on covariables).

^d^ Number of controls included in the analysis (with complete data on covariables).

^e^ Corpus, fundus, Siewert cardia type III.

^f^ Antrum.

### 3.3 The risk of gastric adenocarcinoma by histology

Hypergastrinemia was associated with an increased risk of the intestinal type gastric adenocarcinoma (OR 3.8, 95% CI 1.8‐7.9), but was not statistically significantly associated with the diffuse type (OR 1.6, 95% CI 0.7‐3.7) (Table 3). However, the risk of diffuse type adenocarcinomas was increased in men with hypergastrinemia (OR 3.8, 95% CI 1.0‐14.1), but not in women with hypergastrinemia (OR 0.8, 95% CI 0.3‐2.5). For intestinal type gastric adenocarcinoma, the point estimate was higher in women with hypergastrinemia (OR 6.1, 95% CI 1.8‐20.1) compared to men with hypergastrinemia (OR 2.8, 95% CI 1.1‐7.1). There was no effect modification by age or body mass index (results not shown).

**TABLE 3:** The odds ratio (OR) with 95% confidence interval (CI) of gastric adenocarcinoma with hypergastrinemia (serum gastrin >60 pmol/L) by Laurens histological type and sex

	Both sexes								Women				Men			
	Model 1^a^				Model 2^b^				Model 2^b^				Model 2^b^			
	Cases (No.)^c^	Ctrls (No.)^d^	OR	95% CI	Cases (No.)^c^	Ctrls (No.)^d^	OR	95% CI	Cases (No.)^c^	Ctrls (No.)^d^	OR	95% CI	Cases (No.)^c^	Ctrls (No.)^d^	OR	95% CI
Diffuse	59	111	1.3	0.6‐2.8	59	111	1.6	0.7‐3.7	30	56	0.8	0.3‐2.5	29	55	3.8	1.0‐14.1
Intestinal	72	137	3.4	1.8‐6.4	72	137	3.8	1.8‐7.9	34	63	6.1	1.8‐20.1	38	74	2.8	1.1‐7.1

^a^ Matched for sex, age and sample time.

^b^ As Model 1 and adjusted for body mass index, tobacco smoking status and comorbidity.

^c^ Number of cases included in the analysis (with complete data on covariables).

^d^ Number of controls included in the analysis (with complete data on covariables).

## 4 DISCUSSION

In this prospective population‐based study, hypergastrinemia was associated with an increased risk of gastric adenocarcinoma. The risk was markedly increased for proximal, but not for distal adenocarcinomas.

This is the first study that demonstrates a marked association between hypergastrinemia and subsequent development of gastric adenocarcinoma in the gastrin‐responsive, proximal stomach in a larger general population. However, others have previously found that hypergastrinemia is associated with an increased risk of noncardia gastric adenocarcinoma in a study among smokers,^31^ and we have previously reported that gastric adenocarcinoma patients with hypergastrinemia at the time of diagnosis more often had carcinomas located in the corpus or fundus (81.8% in hypergastrinemic patients vs 36.2% in normogastrinemic patients, *P* = .002).^25^ Numerous animal studies have demonstrated that gastric neoplasia in the corpus develops in models of hypergastrinemia with either reduced, unaltered or increased gastric acidity, strongly suggesting that hypergastrinemia is a causative factor in gastric carcinogenesis and not an epiphenomenon.^17^, ^18^, ^19^, ^20^, ^21^, ^22^, ^23^, ^24^, ^32^ Moreover, patients homozygous to an inactivating mutation of the alpha subunit of the gastric proton pump, which represent a human genetic model of long‐term PPI use, develop hypergastrinemia and gastric neoplasia, including intestinal type adenocarcinoma in the proximal stomach.^33^, ^34^ The risk of gastric neoplasia is increased in *H pylori* infected patients with atrophy of the gastric oxyntic mucosa, but not in patients with *H pylori* infection confined to the antral mucosa, and duodenal ulcer seems to protect against gastric adenocarcinoma.^35^, ^36^ It has therefore been proposed that the carcinogenic effect of *H pylori* infection that causes atrophy of the oxyntic mucosa may be mediated by gastrin.^24^ In theory, an antral tumor of advanced stage could destroy the gastrin releasing antral G cells, giving lower serum gastrin levels in cancers with antral location. However, tumors of advanced stage in the antrum would usually give symptoms that probably would lead to investigations revealing the diagnosis within a short period of time. In the present study, only 7 of the 181 included cases was diagnosed within 1 year of serum sampling and the median follow‐up time from serum sampling to cancer diagnosis was 5.7 (IQR 3.0‐9.1) years. Thus, it is unlikely that advanced stage and tumor destruction of the antral G cells could explain the association found in the present study using prediagnostic serum gastrin measurements.

Laurén's diffuse and intestinal type gastric adenocarcinoma differ in many aspects, including epidemiology, etiology, genetic alterations and molecular signatures.^5^, ^37^, ^38^ These histological subtypes also differ in response to chemotherapy, where patients with diffuse type adenocarcinomas respond less to commonly used regimens and have shorter survival.^39^, ^40^ The current study found that hypergastrinemia was strongly associated with an increased risk of the intestinal type adenocarcinomas, but not with the diffuse type. This is a novel finding in humans, but many of the tumors observed in animal models of hypergastrinemia could also be characterized as intestinal type carcinomas. We also found that women with hypergastrinemia had a higher point estimate of intestinal type adenocarcinoma than men, and that men with hypergastrinemia had a borderline association with diffuse type adenocarcinoma, not found in women. Gender differences are related to many aspects of gastric cancer, exemplified by the marked association between women and diffuse type adenocarcinomas.^41^, ^42^ These possible gender differences should be examined in larger study populations. Previously, higher fasting gastrin levels as well as more pronounced gastrin elevation during PPI therapy or *H pylori* infection have been described in females.^43^, ^44^ However, sex differences in response to hypergastrinemia have not been described in humans, whereas there is a marked female propensity for developing gastric neuroendocrine tumors in rats after long‐term PPI administration.^19^

Strengths of our study included the prospective population‐based design, which counteracted recall and selection bias. Because the exposure was measured in prediagnostic samples years before the gastric adenocarcinoma was diagnosed, the risk of reversed causation was reduced. The complete and high‐quality data on both the exposure and the outcome reduced misclassification and enabled complete follow‐up of the participants. The assessment and adjustment for other risk factors reduced potential confounding. However, in this observational study, residual confounding cannot be ruled out. In our study, we did not have available information on the factors that could cause hypergastrinemia, for example, *H pylori* infection, autoimmune gastritis or use of PPIs. However, regardless of the cause of hypergastrinemia, the present study strongly supports the hypothesis that hypergastrinemia mediates the development of gastric adenocarcinoma in the proximal stomach. Finally, because of the limited sample size, the statistical power was too low for some subgroup analyses.

In conclusion, this prospective population‐based study showed strongly increased risk of gastric adenocarcinoma in the proximal stomach in hypergastrinemic individuals. This supports the hypothesis that hypergastrinemia mediates development of gastric adenocarcinoma in the proximal stomach, where mucosal proliferation is stimulated by gastrin.

## CONFLICT OF INTEREST

The authors declare no conflict of interest.

## ETHICS STATEMENT

At participation, the HUNT participants gave a broad written consent to future research, including linkage to registries and medical records. The current study was approved by the Reginal Committee for Medical and Health Research Ethics, South‐East (reference number 2016/112).

## Data Availability

The research data used in this study is provided by the Nord‐Trøndelag Health Study, the Cancer Registry of Norway and the Norwegian Patient Registry. The data are available through applications to the providers.
